# The Need for Standards Unification in Forensic Laboratory Practices: Protocol for Setting Up the Arab Forensic Laboratories Accreditation Center

**DOI:** 10.2196/36778

**Published:** 2022-06-29

**Authors:** Maha K AlMazroua, Naglaa F Mahmoud

**Affiliations:** 1 Regional Poison Control Center Ministry of Health Dammam Saudi Arabia; 2 Forensic Medicine and Clinical Toxicology Department Faculty of Medicine Cairo University Cairo Egypt

**Keywords:** forensic, practice, Arab countries, accreditation, standards, protocol, best practices, laboratories

## Abstract

**Background:**

Despite the recent trend toward developing and applying quality control measures in the forensic science disciplines, there is a heightened interest to efforts to ameliorate disparate quality among laboratories through the enforcement of standards and best practices.

**Objective:**

This protocol aims to set up the Arab Forensic Laboratories Accreditation Center (AFLAC) to act as a key driving legal force for accreditation of the different forensic laboratories in the Arab region.

**Methods:**

Upon its development, the Forensic Laboratory-Arabian Gate (FLAG) platform will serve as a preliminary stage for the AFLAC, and 2 preparatory steps will be achieved through the FLAG platform: the first one is a scoping study to analyze the international guidelines regarding the forensic laboratory practices in different specialties, and the second one is mapping surveys to explore how the international and national guidelines are translated into practice in Arab forensic laboratories. Development of the Arab forensic laboratories accreditation center will be initiated by building the AFLAC quality management system, which comprises formation of the forensic science committees to achieve the standards required for accreditation in each discipline. This will be followed by the attainment of regional accreditation recognition of the Arab Accreditation Cooperation (ARAC) and the International Laboratory Accreditation Cooperation. This recognition necessitates achieving International Organization for Standardization/International Electrotechnical Commission 17011 standard requirements prior to official application to the ARAC.

**Results:**

The first phase of our work (the FLAG platform) began in February 2022 and is expected to end in December 2022. The FLAG platform was proposed to the Arab Society of Forensic Sciences and Forensic Medicine in March 2022, and we received approval to host this web development project. Subsequent phases are anticipated to begin in January 2023 and are expected to end in 2025.

**Conclusions:**

This work describes our approach to provide a valuable tool for forensic laboratory accreditation services to promote the best practice and its consistency in the field of forensic sciences in the Arab region.

**International Registered Report Identifier (IRRID):**

PRR1-10.2196/36778

## Introduction

The term forensics has been linked to many different branches: economics, anthropology, engineering, dentistry, psychology, pathology, toxicology, entomology, accounting, and computer forensic science [1]. There are several disciplines of forensic science including, but not limited to, forensic biology; forensic chemistry, including seized drugs and postmortem toxicology, and forensic chemistry; trace evidence; digital or multimedia forensic science; forensic medicine; forensic physics or pattern interpretation; and forensic scene examination [1].

Forensic science is a highly specialized area that mandates the collaboration of the forensic community in Arab countries to utilize forensic technologies and techniques to solve crimes, investigate deaths, and protect the public [2,3].

Several challenges are faced by the forensic community, including assortment (standard operating procedures, methods, resources, and oversight); lack of mandatory standardization, certification, and accreditation; the extent and diversity of forensic science branches; the interpretation of forensic evidence (such as the validity of the various disciplines and the degree of scientific research); the need for measures of performance; and the use of forensic evidence in legal action [4,5].

These challenges definitely pose a continuing and serious threat to the quality and truthfulness of forensic science practice. it is evident that change and advancements, both systemic and scientific, are necessary to ensure reliability, exchange experiences, establish enforceable standards, and promote best practices and their consistency in the field of forensic sciences [6,7]. Establishing standards for the accreditation of forensic science laboratories and the certification of forensic scientists and medical examiners or forensic pathologists play a vital role in promoting the development of forensic science and achievement of best practices [8].

In the forensic arena, there are multiple organizations around the world that provide accreditation to the International Organization for Standardization (ISO), using the standards of 17020 and 17025, which together encompass the forensic investigation process from the scene to the court. Accreditation represents assurance given to the judicial system and the confidence given to the public that a quality standard has been followed and will be maintained throughout the process. It includes the policies, procedures, and practices with method validation and staff competence. Proper application of a quality system, which is mandatory to achieve the standards required for accreditation, will detect potential problems at the individual and systemic levels [9].

The Arab Accreditation Cooperation (ARAC) is the regional cooperation body, which aims at coordinating and developing the accreditation infrastructure in the regional Arab countries. The ARAC is officially incorporated as the regional accreditation cooperation body for the region with a developed quality management system including all procedures, guidelines, and forms following the International Laboratory Accreditation Cooperation (ILAC) and the International Accreditation Forum (IAF). The ILAC is the international organization for accreditation bodies operating in accordance with the ISO 17011:2004 conformity assessment and ILAC and IAF requirements [10,11].

Our aim is setting up the Arab Forensic Laboratories Accreditation Center (AFLAC) to act as key driving legal force for accreditation of different forensic laboratories in the Arab region. This will be proceeded by the development of the Forensic Laboratory-Arabian Gate (FLAG) platform.

## Methods

### Phase 1

#### Overview

Development of the FLAG platform will serve as a preliminary stage for the AFLAC. The aim of the FLAG platform is to represent the scientific, academic, and professional network of the Arab forensic community members by allowing discussing, sharing, and exchanging of ideas regarding scientific analytical methods, protocols, and guidelines related to forensic laboratory practices.

There are 2 preliminary steps to achieve through our FLAG platform: the first one is to analyze the international guidelines regarding the forensic laboratory practices in different specialties (a scoping study) and the second one is to explore how the international and national guidelines are translated to practice in Arab forensic laboratories (mapping surveys).

#### Participants

Membership to the FLAG platform is achieved through election and is open to all individuals who possess sufficient appropriate experience or qualifications relevant to, or those who can show a constructive interest in, the forensic sciences.

The identification of experts in different forensic medicine specialties in the Arab region will carried out using the following strategies: announcement and distribution of the FLAG platform and calls for membership, and direct communication with state or public institutes, university departments, authors of on-topic publications, and national professional associations. Different forensic sciences committees will be constituted and will be invited to the scoping study, mapping surveys, and developments of the standards and guidelines for best practices in their respective relevant specialties.

The scoping study aims to analyze the international and national guidelines on forensic science practices. It includes guidelines approved by international and national professional associations or national toxicologist working groups, in addition to documents published by national authorities or international organizations regarding scientific or expert recommendations [12]. For example, search strategies for relevant documents in the field of forensic toxicology have been developed between January and March 2022 using the following sources: PubMed, Web of Science, and Google with the search keys “forensic toxicology” (to derive guidelines or standards for forensic practice) and “drugs” OR “drugs of abuse” OR “illegal drugs” OR “drug-related deaths” OR “poisoning and forensic chemistry services”; a targeted document search on the websites of The International Association of Forensic Toxicologists, the Arab Scientific Working Group of Forensic Toxicology, the Arab Society for Forensic Sciences and Forensic Medicine, International Association of Forensic Toxicologists, the Scientific Working Group for Forensic Toxicology, the Nordic Association of Forensic Toxicologists, and the Society of Forensic Toxicologists [13-15].

#### Surveys of Practices: Mapping the Practices Implemented in Each Country

The objective of these expert surveys was to analyze how international and national guidelines regarding forensic investigations are translated into practice in forensic laboratories in Arab countries.

#### Data Collection

Forensic practitioners and experts representing different forensic specialties will be invited to complete a series of surveys (specific for each discipline generated by the relevant committee); for example, the forensic toxicology survey (see Multimedia Appendix 1). Experts on toxicological analysis based in laboratories undertaking postmortem analyses will be contacted and asked to participate in a survey on their laboratory performance in suspected drug-related deaths, guidelines and standards for laboratory practice and result reporting, analytical strategies, technical equipment, equipment validation, laboratory quality control principles, and potential hindrances or challenges to their daily work on drug-related deaths. Finally, based on these results, some general conclusions will be drawn and potential implications discussed with regard to how to interpret drug-induced deaths and prevalence data, taking into consideration the background of toxicology standards and capacities in different countries.

#### Results Analysis

Data collected from the mapping surveys will be analyzed in comparison to the recommended national and international guidelines to evaluate the actual practice and to strengthen areas of defects, if present.

### Phase 2: Setting up of the AFLAC

#### Overview

For seeking international recognition, the AFLAC is required to develop their operating policies and procedures and ensure that its structure conforms to the requirements specified in the international standard ISO/International Electrotechnical Commission (IEC) 17011 standard, “Conformity assessment - General requirements for accreditation bodies accrediting conformity assessment bodies,” and other IAF and ILAC criteria and for regional recognition by the ARAC.

A number of mandatory documents of the IAF and ILAC contain mandatory provisions for policies and procedures, and guidelines for the delivery of the accreditation services must be implemented. In general, the ISO/IEC 17011 standard specifies requirements with respect to impartiality, competence and experience of the staff, management systems, accreditation processes and assessment practices, on-site assessment, surveillance visits, complaints and appeals, and contractual requirements between the accreditation body and its accredited bodies [16].

#### Key Milestones of the FLAG/AFLC Project

The milestones will include announcement and distribution of the FLAG platform as a scientific platform for forensic scientists, medico-legal experts, and academics, allowing their memberships; mapping survey data collection, analysis, and result declaration; establishment of the AFLAC management system and committees for different forensic disciplines; regional accreditation recognition through regional cooperation with the ARAC; development of regional Arab guidelines and AFLAC standards for different forensic science disciplines; distribution and implementation of the AFLAC; and international recognition of the AFLAC by the ILAC and the IAF.

## Results

The first phase of our work (the FLAG platform) began in February 2022 and is expected to end in December 2022. The FLAG/AFLAC platform was proposed to the Arab Society of Forensic Sciences and Forensic Medicine in March 2022, and we received approval to host web development projects on it. Subsequent phases are anticipated to begin in January 2023 and end in 2025.

## Discussion

The vast majority of Arab forensic labs are lacking in the resources (money, staff, training, and equipment) necessary to promote and maintain strong forensic science laboratory systems [17,18].

The result, depth, reliability, and overall quality of standing information and the results arising from the forensic laboratories vary strongly across Arab countries. Operational principles and procedures for many forensic science disciplines in forensic laboratories are not standardized, which further compound such a fragmentation issue. There is no uniformity in the certification of forensic practitioners or in the accreditation of crime laboratories [19-21]. Often, there are no standard protocols governing forensic practice in a given discipline, and even when protocols are in place (eg, scientific working group standards), they often are vague and are not enforced in any meaningful way. In brief, there are several limitations that clearly generate a serious and continuing threat to the quality of forensic science practice, such as the lack of obligatory standardization, certification, and accreditation; the absence of proper training and continuing education; mandatory certification and accreditation programs; commitment to robust performance standards; and effective oversight [21].

Accreditation and certification are both undertaken using third-party bodies, which are, in turn, accredited to perform this service. Many accreditation bodies are signatories to the ILAC. The ILAC is the international organization for accreditation bodies operating in accordance with the ISO/IEC 17011:2004 conformity assessment, which is a general requirement for bodies providing assessment and accreditation of conformity assessment bodies and documents regarding supplementary requirements [22-25].

The Arab region was the only region in the world lacking a regional structure for cooperation in the accreditation field till 2010. Arab countries had no access to support services and training. They have to approach the international accreditation bodies for seeking support, recognition, and training. This process was lengthy and costly and deprived a large number of countries from accessing these services [10]. Furthermore, Arab countries were neither well represented at the international level nor managed to achieve international recognition. Against this backdrop, the United Nations Industrial Development Organization supported a cooperation project for establishment of the ARAC in 2010 as a platform upon which Arab countries can build and develop their accreditation infrastructure. The ARAC has 4 multilateral recognition arrangement signatories: the Egyptian Accreditation Council (EGAC); the Jordanian Accreditation and Standardization System; the GCC (Gulf Cooperation Council) Accreditation Center; and the Emirates International Accreditation Centre [11,12].

Analysis of the accreditation services related to the field of forensic laboratories provided by ARAC members revealed that all of them provide accreditation services only for medical laboratories, while forensic science laboratory accreditation is only provided by the EGAC. The EGAC is the first accreditation body in Arab countries and in the Middle East to begin providing accreditation for forensic service providers, offering accreditation of forensic testing and inspection services in accordance with ISO/IEC 17025 and 17020 standards [10,18].

Areas currently accredited by the EGAC include DNA fingerprinting, autopsy, forensic histopathology, examination of people who have experienced crime, assailants of violence cases, crime scene examination, photography, and radiology. However, forensic toxicology analysis and other forensic services are not covered in the scope of EGAC accreditation. Our aim is to fill this gap by establishing AFLAC to act as key driving legal force for accreditation of the different forensic laboratories in the Arab region [26,27].

Laboratory accreditation and individual certification of forensic science professionals should be mandatory, and all forensic science professionals should have access to a certification process. In determining appropriate standards for accreditation and certification, the AFLAC will consider recognized and established international standards such as those developed by the ISO [28,29].

## Appendix

Multimedia Appendix 1 Mapping survey.

## Abbreviations

AFLAC: Arab Forensic Laboratories Accreditation Center
ARAC: Arab Accreditation Cooperation
EGAC: Egyptian Accreditation Council
FLAG: Forensic Laboratory-Arabian Gate
GCC: Gulf Cooperation Council
IAF: International Accreditation Forum
IEC: International Electrotechnical Commission
ILAC: International Laboratory Accreditation Cooperation
ISO: International Organization for Standardization
