# Correction: The Need for Standards Unification in Forensic Laboratory Practices: Protocol for Setting Up the Arab Forensic Laboratories Accreditation Center

**DOI:** 10.2196/40863

**Published:** 2022-07-14

**Authors:** Maha K AlMazroua, Naglaa F Mahmoud

**Affiliations:** 1 Regional Poison Control Center Ministry of Health Dammam Saudi Arabia; 2 Forensic Medicine and Clinical Toxicology Department Faculty of Medicine Cairo University Cairo Egypt

In “The Need for Standards Unification in Forensic Laboratory Practices: Protocol for Setting Up the Arab Forensic Laboratories Accreditation Center” (JMIR Res Protoc 2022;11(6):e36778) the authors noted one error.

In the originally published article, Reference 6 was incorrectly published as follows:

Naglaa F. The Prevalence of Illicit Drugs and Alcohol in Road Traffic Accident Fatalities in the Eastern Region of Saudi Arabia. IJFMT 2020 Oct 7 [doi: 10.37506/ijfmt.v14i4.12122]

This reference is now corrected as follows:

Mahmoud NF, Al-Mazroua MK, Afify MM. The Prevalence of Illicit Drugs and Alcohol in Road Traffic Accident Fatalities in the Eastern Region of Saudi Arabia. Indian Journal of Forensic Medicine & Toxicology 2020;14(4): 3219-3225. [doi: 10.37506/ijfmt.v14i4.12122]

The correction will appear in the online version of the paper on the JMIR Publications website on July 14, 2022, together with the publication of this correction notice. Because this was made after submission to PubMed, PubMed Central, and other full-text repositories, the corrected article has also been resubmitted to those repositories.
