# Supplementary material for: The Need for Standards Unification in Forensic Laboratory Practices: Protocol for Setting Up the Arab Forensic Laboratories Accreditation Center
Source: JMIR Res Protoc. 2022 Jun 29;11(6):e36778. doi: 10.2196/36778 (PMC9280477; doi:10.2196/36778)
Supplement: Multimedia Appendix 1 [file resprot_v11i6e36778_app1.pdf]

Forensic Toxicology Mapping Survey

1. My laboratory currently held:

- No accreditation certificate
- Clinical Laboratory Improvement Amendments (CLIA) accreditation certificate
- the College of American Pathologists (CAP) accreditation certificate
- the American Board of Forensic Toxicology (ABFT) accreditation certificate

2. The following services are provided by my laboratory:

- Many services; toxicology is one type of service
- Toxicology testing in impaired driving cases
- Clinical toxicology testing
- Postmortem testing
- Human performance
- Criminal justice supervision
- Workplace drug testing
- Substance abuse treatment
- other toxicology services (occupational & molecular toxicology).

3. **The average number of cases in my lab is:**

- 250,000 or more
- 50,000–249,999
- 20,000–49,999
- 10,000–19,999
- 5,500–9,999
- 3,500–5,499
- 1,500–3,499
- 1,000–1,499
- 500–999
- 0–499

4. **Average Turnaround Time to Complete Cases in my lab is:**

- 1-5 days
- 5-10 days
- 10-15 days
- More than 15 days

5. **Always a complete autopsy is done where samples for toxicological analyses are collected**

- Always
- Frequently
- Never
- Sometimes

6. **The following kinds of specimen in use for testing in my lab:**

- Urine
- Serum
- Whole Blood
- other Matrix

Mentions of Other matrices:

- Cerebrospinal fluid
- Vitreous humor
- Bile • Liver • Stomach contents
- Pericardial fluid
- Muscle
- Kidney dialysate

7. **Chromatographically methods are used as screening tests:**

- yes
- no

8. **Routine immunological screening tests related to drugs of abuse are used in my laboratory:**

- yes

- no

9. **Positive immunological screening or other screening test results are routinely confirmed?**

- yes
- no

10. immunological screening or other pre- tests was done the following substance group:

- Barbiturates
- Cannabinoids
- Amphetamine
- Cocaine
- Opiate
- Benzodiazepines

11. Immunological screening or other pre- tests was done for the following specific substance:

- Zolpidem
- Buprenorphine
- Methadone
- Fentanyl
- Tramadol
- Phencyclidine
-

12. **Strategy for analysis in suspicious postmortem cases always "general unknown"**

- yes, always "gen. unknown scr.
- " no, focused/case- dependent
- always "general unknown" AND focused/case-dependent

13. The following laboratory instruments are available in my lab:

- TLC
- Spectrophotometer
- GCMS
- LCMS
- GCMSMS
- HS-FID
- HS-MS
- LCMS-QTOF
- Dry blood spot
- Infrared
- Raman

14. **When questions arise about substances you cannot analyze in your laboratory: Do you have the opportunity to send samples to a specialized lab.**

- yes
- no

15. **Quantitative determination of the major drug(s) that caused death is performed**

- yes
- no

16. Quantitative determination of the other drugs that might have contributed to death is performed

- yes
- no

17. **My final report include quantitative results**

- Always
- Frequently

- Never
- Sometimes

18. My final report include qualitative results

- Always
- Frequently
- Never
- Sometimes

19. **My final report include interpretation of results**

- Always
- Frequently
- Never
- Sometimes

20. **Incidental/ additional findings which are rated as being irrelevant for the cause of death are listed in my report**

- Always
- Frequently
- Never
- Sometimes

21. **What is your level of satisfaction regarding the exchange of case-related information between institutions (police/hospitals/ forensic pathologists) and your laboratory?**

Very satisfied

Satisfied

OK

Dissatisfied

Very dissatisfied

22. My analytical strategy Routinely cover

- Opiates
- Cocaine
- Amphetamine
- Methamphetamin MDMA, MDA, MDEA
- Nat.Cannabin.
- Volatiles Gases(Butane,Prop.)
- Solvents
- Prescr.opoids
- Pesticides
- Carboxyhb
- Alcohols

23. My analytical strategy cover the following substances on request only

- Methadone
- Buprenorphine
- Fentanyl
- Tramadol
- Benzodiazepines
- Drugs
- Carbon monoxide

24. **My analytical strategy cover the following new psychoactive substances(NPS)**

- Synth.Cannabinoids
- Synth.Cathinones
- Designer-opioids
- Designer-Benzod.
- Piperazines
- Phenylethylamines
- Designer Cocaine

25. **The following limitations concerning the toxicological analyses in my laboratory could be:**

- budgetary restraints
- lab equipment
- reference substances

26. I recommend the following suggestions for improving the analytical strategy of my laboratory: • Improve…

- Networking/Communication with other stakeholders (police, justice, health system)
- Cooperation with forensic pathologists
- Standardization of sample collection
- Technical and operational needs: Equipment, methods needed
- Funding for method development and research
- Guidelines for postmortem toxicological analyses
- Standards for forensic laboratories accreditation
